# Importance of High-Risk Human Papillomavirus Infection Detection in Female Renal Transplant Recipients in the First Year after Transplantation

**DOI:** 10.1155/2018/9231031

**Published:** 2018-12-06

**Authors:** Maksims Cistjakovs, Alina Sultanova, Olga Jermakova, Liba Sokolovska, Svetlana Chapenko, Baiba Lesina-Korne, Rafail Rozental, Modra Murovska, Ieva Ziedina

**Affiliations:** ^1^August Kirchenstein Institute of Microbiology and Virology, Riga Stradins University, Riga, Latvia; ^2^Gynecology and Maternity Unit, Pauls Stradins Clinical University Hospital, Riga, Latvia; ^3^Scientific Laboratory of Transplantology, Riga Stradins University, Riga, Latvia

## Abstract

**Objectives:**

Most of human papillomavirus (HPV) infections are “cleared” by the immune system; however, in cases of immune system suppression, infections could lead to development of malignancies. The aim of this study was to find out the frequency of HR-HPV infection in early period after renal transplantation in recipients receiving immunosuppressive therapy and to follow the progression of the infection up to one year.

**Methods:**

43 female renal transplant recipients and 79 healthy female individuals as a control group were enrolled in this investigation. For the detection of HPV infection, patients' samples (blood and vaginal swabs) were collected two weeks after transplantation with following collection of six months and one year. Different polymerase chain reactions for HR-HPV genomic sequences detection and ELISA kit for detection of anti-HPV IgG antibodies were used.

**Results:**

In this study, we show that frequency rate of HR-HPV infection has increased in the first year after transplantation from early stage of immunosuppressive therapy (from 24% to 36%). Also an increase of HR-HPV load was detected over time, showing the highest median viral load at sixth month after transplantation.

**Conclusions:**

From the obtained data, it follows that it is very important to carefully monitor patients receiving immunosuppression therapy on progression of HR-HPV.

## 1. Introduction

Human papillomavirus (HPV) infections remain one of the major global burdens even despite the very active use of vaccination. Prevalence rate of HPV in East Europe is about 21.4% which is much higher in comparison with the global prevalence of 11.7% [1]. Such high prevalence rate could be related to absence of HPV screening programs and low vaccination uptake because of poor knowledge on HPV infection in the population [2, 3].

Based on association with cervical cancer and precursor lesions, HPV can be classified as high-risk (HR-HPV) and low-risk (LR-HPV) oncogenic types [4, 5]. LR-HPV types, such as HPV 6 and 11, can cause common genital warts or benign hyperproliferative lesions with very limited tendency to malignant progression, while infection with HR-HPV types, highlighting HPV 16 and 18, are associated with the occurrence of premalignant and malignant cervical lesions [6].

Most of HPV infections are “cleared” by the immune system and do not result in clinical diseases in healthy individuals; however, in cases of immune system suppression, infections could lead to development of malignancies [7].

Although immunosuppressive therapy has improved long-term graft and patient survival after renal transplantation, it increases the cumulative occurrence of (pre)malignancies, especially those associated with viral infections [8–10]. Declined cell-mediated immunity caused by the use of immunosuppressive therapy could increase risk for HPV related anogenital (pre)malignancies in renal transplant recipients, especially in countries where the prevalence of HPV is high. Previous studies have shown dramatic increase of HR-HPV infection up to 27% [11, 12].

*The objective* of this study was to find out the frequency of HR-HPV infection in early period after renal transplantation in Latvian recipients and to follow the progression of the infection up to one year.

## 2. Methods

43 female renal recipients (median age of 48; IQR = 39-58), who received kidney allograft during 2013-2015, and 79 healthy female individuals (median age of 48; IQR = 42-57), who were visiting gynaecologist for preventive examination, as a control group were enrolled in this investigation. For the early detection of HPV infection, patients' samples (whole blood and vaginal swabs) were collected two weeks after transplantation with following collection of six months and one year after the transplantation to receive data on later periods. Chronic glomerulonephritis (7%), hypertensive nephropathy (21%), chronic interstitial nephritis (26%), and polycystic kidney disease (26%) were the most common reasons for the subsequent transplantation. All patients had received induction immunosuppression therapy with monoclonal or polyclonal antibodies and steroid bolus course. Initially immunosuppressive therapy consisted of glucocorticoids (Prednisolone tapered down to 5 mg per day during study period), antiproliferative drugs (Cell-cept ® 2 g per day, tapered down to 1g per day if leucopenia appeared), and calcineurin inhibitors (once per day tacrolimus with trough level of 7-10 ng/ml during first 3 months after surgery and 5-8 ng/ml thereafter or microemulsified formulation of cyclosporine with trough level of 150-250 ng/ml during first 3 months after surgery and thereafter 100-200 ng/ml for patients transplanted in 2013).

Aliquots of 200 *μ*l blood plasma were collected from EDTA peripheral blood samples for further serological tests. Blood plasma samples and cervical swab samples were stored at -70°C.

DNA from cervical swab samples was extracted using phenol-chloroform method.

Beta- (*β*-) globin PCR with appropriate primers was used to determine the quality of isolated DNA [13].

Polymerase chain reaction (PCR) with consensus primers MY9/MY11 was used for initial detection of high range HPV types (HR-HPV and LR-HPV types) [14], HPV High Risk Screen Real-TM Quant commercial qPCR kit (Sacace, Italy), for quantitative detection of 12 types of HR-HPV (16, 18, 31, 33, 35, 39, 45, 51, 52, 56, 58, and 59), and HPV typing with semiquantitative viral load determination was done with the Anyplex™ II HPV28 kit (Seegene, South Korea) in recipients' cervical swab DNA samples.

ELISA commercial kit (MyBioSource, USA) was used to detect IgG class antibodies against HR-HPV L1-capsids' protein in recipients' plasma (presence of these antibodies is associated with HR-HPV clearance) [15, 16].

All statistical calculations and graphs were performed using GraphPad Prism software version 6.0 for Windows (GraphPad Software, San Diego, CA, USA). To test whether the collected data are normally distributed, the D'Agostino and Pearson, Anderson-Darling, and Shapiro-Wilk normality tests were applied. Statistical differences in the prevalence of HPV infection were assessed by using Fisher's exact test. Statistical differences in viral load comparison were calculated by Mann–Whitney U test. Because most of the data was distributed not normally, results are expressed as median and interquartile range (IQR) as dispersion characteristic, and p-value of less than 0.05 (p< 0.05) was considered as statistically significant. Pearson and Spearman tests were used for correlation analysis.

The study was approved by the Ethics Committee of Riga Stradins University, and written consent was obtained from all patients.

## 3. Results

### 3.1. Presence of HPV Sequences Detected by PCR with MY9/11 Consensus Primers

#### 3.1.1. Recipients

Initial PCR results with consensus primers MY9/11 showed that almost all recipients (42/43; 98%) were positive on HPV genomic sequences in cervical swab DNA samples. In 28/43 (65%) recipients' cervical swab samples, viral DNA was detected two weeks after transplantation, in 25 samples viral DNA had been preserved, and in 8 (total 33 recipients; 77%) additional recipients' presence of HPV genomic sequences in cervical swab DNA samples appeared (total 33/43 recipients; 77%) six months after transplantation. One year after transplantation, 24 recipients were still positive on HPV genomic sequences in cervical swab DNA samples (17/24 were positive after two weeks and 6 months after transplantation and another 7 recipients were positive only after a time period of 6 months).

#### 3.1.2. Control Group

Frequency rate of HPV sequences in control group's cervical swab DNA samples was found to be significantly lower (38%  [30/79] vs 65%  [28/43, two weeks], 77%  [33/43, six months], and 72%  [31/43, one year], p< 0.05).

### 3.2. Presence of HR-HPV Sequences Detected by Two Commercial Real-Time PCR Kits

#### 3.2.1. Recipients

All HPV positive samples were tested on the presence of HR-HPV types using the Anyplex™ II HPV28 kit (Seegene, South Korea) and viral load was detected using HPV High Risk Screen Real-TM Quant commercial qPCR kit (Sacace, Italy).

**Figure 1:**
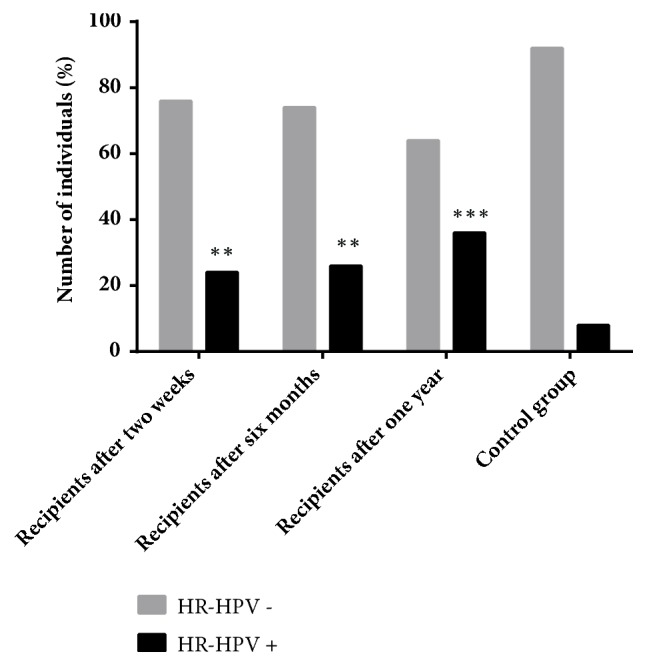
Presence of HR-HPV genomic sequences in recipients' group cervical swab DNA samples two weeks, six months, and one year after transplantation in comparison with control group. *∗*p value in comparison with control group.

Overall HR-HPV genomic sequences were found in 65% (28/43) of recipients. In early period after transplantation (two weeks), HR-HPV presence was detected in 24% (10/43) of recipients. In the following time periods, six months and one year, HR-HPV frequency increased till 26% and 36%, respectively (Figure 1). McNemar's test showed no significance in HR-HPV positivity frequency over time in the kidney transplant group. However, comparison of each recipient's time points with control group showed significant higher HR-HPV frequency in patients over the whole study period (one year after transplantation vs control group, p=0.0001; OR= 1.778, 95% CI: 1.322-2.407)

**Figure 2:**
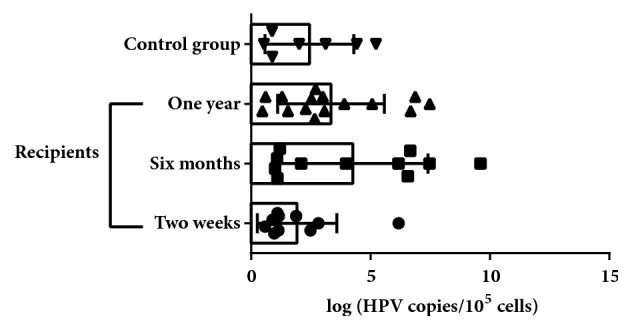
HR-HPV median log copies detected in recipients' group cervical swab DNA samples two weeks, six months, and one year after transplantation in comparison with control group.

The highest HR-HPV median load was detected on the sixth month after transplantation in comparison to two-week and one-year period (3.98 log copies/10^5^ cells, IQR: 1.10-6.66 versus 1.15 log copies/10^5^ cells, IQR: 0.95-2.57, and 2.70 log copies/10^5^ cells, IQR: 1.55-5.07 [Figure 2]).

**Figure 3:**
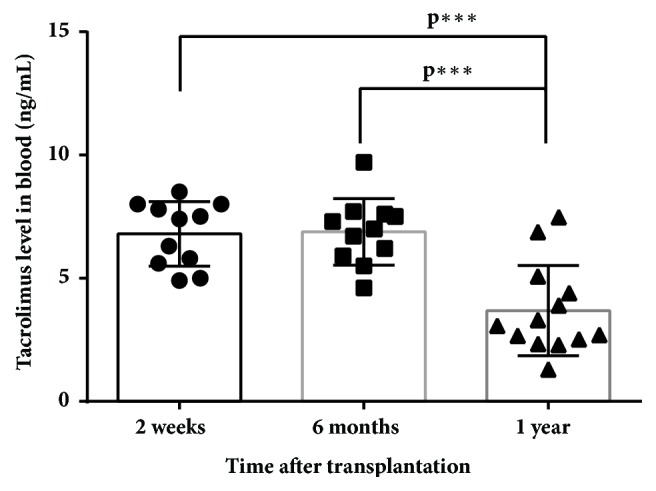
Average levels of tacrolimus in recipients' peripheral blood positive for HR-HPV.

Although significant differences in HR-HPV load were not found between recipients in the different time points, decrease of median viral load after one year coincides with significant decrease of average tacrolimus level in the same period in comparison to 2 weeks and 6 months after transplantation (3.68±1.83 ng/mL against 6.80±1.31 and 6.88±1.35 ng/mL, resp.) (Figure 3).

Following HR-HPV types were detected among the recipients: 16, 18, 31, 33, 35, 51, 56, 66, 68, and 73. The most dominant detected type was HPV-18, in 14/42 recipients (33%), in comparison with HPV-56 (8/42, 19%), HPV-16 (7/42, 17%), HPV-35 (7/42, 17%), HPV-51 (6/42, 14%), HPV-68 (5/42, 12%), HPV-33 (3/42, 7%), HPV-73 (2/42, 5%), HPV-66 (1/42, 2%), and HPV-31 (1/42%, 2%), however without statistical significance.

**Figure 4:**
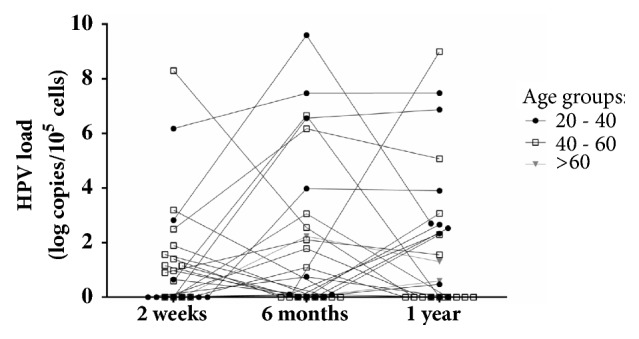
HR-HPV load (log copies/10^5^cells) detected in each recipient's cervical swab DNA samples two weeks, six months, and one year after transplantation.

Patients' data was stratified in three age groups. Patients corresponding to the age group of 20 – 40 years showed higher HR-HPV loads in their samples six months after transplantation in comparison to patients of age over 40 years (Figure 4).

**Figure 5:**
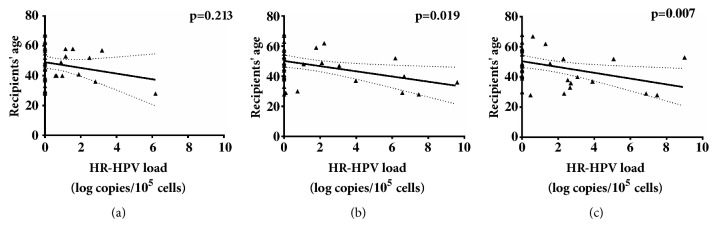
HR-HPV load (log copies/10^5^cells) and age correlation two weeks (a), six months (b), and one year (c) after transplantation.

Statistical analysis showed correlation between age and HR-HPV load in a period of six months and one year after transplantation (Figure 5).

#### 3.2.2. Control Group

Among control group, HR-HPV infection was determined in 7/79 (8%) of women, significantly lower frequency than in recipients' group (two weeks, 23%; p=0.05, six months, 26%; p=0.01, and one year, 36%; p<0.01).

Even though median HR-HPV load in control group was higher than in recipients' group two weeks after transplantation (2.01 log copies/10^5^ cells, IQR: 0.88-4.43 vs 1.15 log copies/10^5^ cells, IQR: 0.95-2.57, resp. [Figure 2]), there was no significance.

The following HR-HPV types were detected: 16, 31, 33, 45 and 59.

### 3.3. Presence of IgG Antibodies against HR-HPV

IgG antibodies against HR-HPV were detected in 15/43 (35%) recipients, where 10 recipients were positive during all three checking periods, two recipients only were positive at two-week period, another one was positive during the first two periods and negative at one year after transplantation, and one recipient was positive during the last two periods and one recipient was positive only one year after transplantation. Two recipients among those who became negative on HR-HPV IgG antibodies were receiving Rituximab, B lymphocytes' suppressor.

Plasma samples received from control group showed significantly higher frequency rate of antibodies against HR-HPV (51/79, 65%, p<0.001).

**Figure 6:**
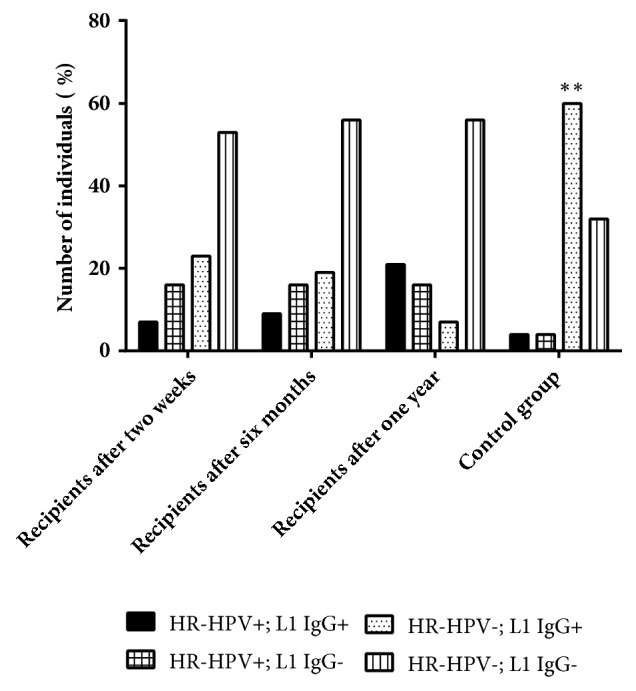
Comparison of HR-HPV genomic presence in cervical swab DNA samples together with presence of anti- HR-HPV L1 IgG antibodies in recipients' group of different testing time points compared with control group (*∗*p value).

Interestingly, the number of the recipients positive for both, HR-HPV sequences and antibodies, was increasing over the time after transplantation and the number of recipients positive for antibodies only decreased (Figure 6).

### 3.4. Allograft Rejection Cases

During the study, 11 out of 43 (26%) recipients developed different types of allograft rejections, the most frequent of which was acute humoral rejection (5 out of 11 cases) (Table 1). HR-HPV infection was detected in 8 out of 11 (72%) recipients with rejection. Viral infection was more frequently found in recipients with acute humoral allograft rejection 2 weeks after transplantation (in 4 out of 5 recipients) (Table 1). Clinically significant HR-HPV load (>3 log copies/10^5^ cells) was found relatively more frequently (3 out 8; 38%) in recipients with rejection in comparison to recipients with normal allograft function, where clinically significant viral load was found in 6 out of 20 (30%) recipients, although without statistical significance.

### 3.5. Histological Results

**Figure 7:**
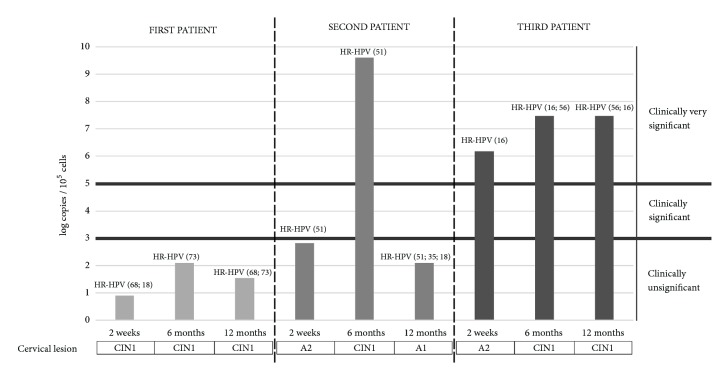
Progression of CIN development in three recipients over the time in comparison to HR-HPV load with qPCR result interpretation provided by manufacturers' protocol (Sacace, Italy).

Presence of cervical intraepithelial neoplasia (CIN) in one recipient was detected on the second week after transplantation. After six months, two more recipients had developed CIN I, and after one year, one of the three recipients with CIN I cytology results were normal. In all DNA samples obtained from cervical swabs of recipients with CIN high HR-HPV loads were detected and in two of them coinfection with several HPV types was found (on the second week, 1st recipient: HPV-68+HPV-18; 0.9 log copies/10^5^ cells; on the sixth month, 1st recipient: HPV-73; 2.1 log copies/10^5^ cells, 2nd recipient: HPV-51; 9.6 log copies/10^5^ cells, and 3rd recipient: HPV-16+HPV-56; 7.47 log copies/10^5^ cells; after one year, 1st recipient: HPV-68+HPV-73; 1.55 log copies/10^5^ cells, 2nd recipient: HPV-51+HPV-35+HPV-18; 2.7 log copies/10^5^ cells [cytology results: normal], and 3rd recipient: HPV-16+HPV-56; 7.48 log copies/10^5^ cells) (Figure 4). Antibodies against HR-HPV among these three recipients during all three testing points were detected only in the 1st recipient's plasma samples, and the other two recipients were antibody negative (Figure 7).

Cytology results of other recipients had shown normal epithelium or such pathologies like atrophy, colpitis, and cervicitis.

Among control group individuals, no CIN cases were detected; however, one individual with very high HPV-16 load had atypical squamous cells of unknown significance (A2-ASCUS).

## 4. Discussion

HPV persistent infection is strongly associated with development of cervical cancer [17]. Despite the development of new vaccines and increased use of them worldwide, HR-HPV infections remain one of the major global health burdens. This could be related to several circumstances like low uptake of vaccine in some countries and absence of HPV screening programs, as well as the fact that vaccines do not provide defense from all HPV types. In Latvia, HPV vaccination as a routine immunization approach was included in the National Health Care Program in 2010; however, uptake of the vaccine is not sufficient [3, 18].

The HPV clearance from host's organism requires an adequate immune response; therefore, immunocompromised individuals could be in the risk group of HPV persistent infection development [19]. Because of received immunosuppressive therapy, renal allograft recipients in particular are at high risk of developing persistent infection and HPV associated cancer [20]. Recently published cohort study from Denmark shows fivefold increased risk of genital warts (GW) development associated with HPV infection in female kidney transplant recipients in comparison with a nontransplanted cohort [21]. Also, they showed a tendency of lower risk of GW development in recipients that are undergoing dialysis procedure due to graft failure after transplantation in comparison to recipients with normal graft function [21]. These results also coincide with the results of another cohort study from Australia, where the same tendency was shown only in the development of anogenital cancer in the recipients [22]. This could be explained by chronic inflammation caused by the exposure of recipients to uremic toxins and contact with the dialysis system [23]. It was shown that dialysis could have a dramatic impact on the innate immune response, especially on macrophage activity, which is important because the innate immune response plays a crucial role in HPV clearance from the hosts' organism preventing its persistence [23, 24]. However, our study found no significant differences between recipients with transplant rejection and recipients with normal transplant function in HR-HPV infection presence (72% vs 62%, resp.) and high viral load frequencies (38% vs 30% with >3 log copies/10^5^ cells, resp.).

Another recent study showed that papillomatous lesions in the oral cavity occurred in 36.1% of kidney allograft recipients within 2 years after transplantation, where 25% of recipients were positive on HPV-16 DNA sequence. More than half of the studied group were male recipients [25]. This shows that HPV infection creates risk for not only cervical cancer development in female recipients, but also HPV associated oral cancer which could be developed in both male and female recipients.

That means a broader study on different HPV associated cancer type developments, including anal cancer development, whose incidence has been rising in the last few years, is needed [26]. Such research would require not only female but also male recipients' study groups, because men are not vaccinated against HPV and could be in higher risk of oral and/or anal cancer development.

Another crucial factor that has an effect on the HPV infection is the median age, 48, IQR= 39-58, of enrolled recipients. This means that they were not vaccinated, because, as was written above, HPV vaccination was included in Latvian National Health Care Program in 2010 and should be done before the age of 15. Preventive vaccination against HPV could help decrease HPV related cancer development in the future; however, vaccination in adulthood is not very efficient, especially in the case of immunosuppressant, due to the fact that, after the first infection, HPV may persist or be integrated in the hosts' genome and therefore escape from humoral immune response. Therefore, recipients, who missed vaccination or were not able to be vaccinated during childhood, are in the highest risk group of developing HPV associated cancers. New ways to manage HPV infection should be found.

The results of this research show a significantly higher frequency rate of HR-HPV infection in renal transplant recipients even in the early period after transplantation (two weeks, 24%; p=0.05, six months, 26%; p=0.01, and one year, 36%; p<0.01) in comparison with control group (8%). These results show the growing tendency of HR-HPV infection frequency over the time in female recipients receiving immunosuppressive therapy which indicates they are in the risk group for HPV persistent infection development and possible development of cervical cancer.

The fact that the highest level of HR-HPV load was detected in recipients' DNA samples six months after transplantation in comparison with control group (median 3.98 log copies/10^5^ cells, IQR: 1.10-6.66 against 2.01 log copies/10^5^ cells, IQR: 0.88-4.43) is another evidence showing immunosuppression as a high-risk factor of development of HPV persistent infection. The increase of HR-HPV viral load detected in recipients' samples from two weeks to six months (from 1.15 log copies/10^5^ cells, IQR: 0.95-2.57, to 3.98 log copies/10^5^ cells, IQR: 1.10-6.66, p<0.11) could be related to the deepening of immune system suppression because the mean CD4/CD8 ratio dropped from 1.95±1.07 till 1.51±0.87. However, it is not clear why median viral load has started to decrease in the one-year period after transplantation (till 2.70 log copies/10^5^ cells, IQR: 1.55-5.07). It may be related to the lowering of immunosuppressive drug dosages as significant decrease of tacrolimus in recipients' peripheral blood one year after transplantation in comparison to 2 weeks and 6 months after transplantation (3.68±1.83 ng/mL against 6.80±1.31 and 6.88±1.35 ng/mL, resp.) (Figure 3) was found in this study.

Significant correlation between the age of recipients and HR-HPV load was found only in the period of six months and one year after transplantation (Figure 5). This again indicates that immunosuppressive therapy has major impact on HR-HPV infection progression, as in these periods after transplantation, recipients' immunosuppression reaches maximum effect. Also, recipients in the age ranges between 20 and 40 years were found to have higher viral loads. This together with the correlation results indicates that this group could be in higher risk of HR-HPV persistent infection development which plays an important role in cervical cancer development (Figures 4 and 5).

Higher frequency rate of IgG antibodies against HR-HPV L1 capsid protein in control group rather than in recipients' group (65% vs 35%, p=0.001) is another evidence that immunosuppression plays an important role in the development of persistent HR-HPV infection. Also, in some recipients who had received Rituximab, which inhibits B lymphocytes' activity, no detectable antibodies against HR-HPV in the early period after transplantation were found. This could indicate the crucial role of immunosuppressive therapy, including Rituximab, in suppression of humoral immune response against HPV infection.

Interestingly, 14% of recipients who were previously negative for the presence of HR-HPV genomic sequences but positive for the presence of antibodies against HR-HPV L1 protein became positive for viral sequences in the next observation period after transplantation (Figure 6). This could indicate HR-HPV reactivation and support hypothesis that HPV could have a latent phase. It is still not clear how HPV creates latency, research using mouse models have shown that as long as the virus remains in the basal layer's stem cells, viral replication does not occur, but HPV can become active in differentiated cells [27]. In the case of HPV latent infection, presence of virus-specific antibodies could not be considered as a marker of HPV clearance [28]. To evaluate development of HPV infection, more specific markers which could indicate HPV latency need to be found.

Presence of cervical intraepithelial neoplasia is detected only in recipients' group and in all three cases HR-HPV infection was found. Also, high viral loads were detected in those patients (9.6, 7.48, and 2.1 log copies/10^5^ cells). In these cases, two facts should be pointed out: (1) in one recipient with high HPV load (9.6 log copies/10^5^ cells) six months after transplantation clearance of CIN I followed with viral load decrease to 2.7 log copies/10^5^ cells was found after the one-year period; (2) another recipient showed presence of CIN I during all three testing points with low viral load (0.9, 2.1, and 1.55 log copies/10^5^ cells) and was also positive for antibodies against HR-HPV. The first fact shows that HR-HPV load could potentially be a useful marker in the progression of HPV associated CIN development. The second fact points out the presence of antibodies as a positive marker which indicates HPV load decrease.

In this study, we show that frequency rate of HR-HPV infection has increased in the first year after transplantation from early stage of immunosuppressive therapy (from 24% to 36%). Together with increase in viral load (highest median is detected on sixth month after transplantation, 3.98 log copies/10^5^ cells, IQR: 1.10-6.66), these results indicate that female renal transplant recipients are at high risk of developing HPV associated cervical intraepithelial neoplasia of different grade and possibly even cervical cancer. Therefore, all female recipients need to be more carefully monitored (twice a year during the first two years after surgery) for HPV infection and development of CIN from the beginning of immunosuppressive treatment application. The presence of several cases of CIN I in recipients' group but not in control group could support this statement. Early detection of HR-HPV infection in female renal transplant recipients could help prevent development of cervical cancer in the future.

## Figures and Tables

**Table 1:** Number of recipients with different types of allograft rejection.

**Allograft rejection types**	**Number of recipients (n=11)**	**Time after transplantation**	**Number of recipients positive on HR-HPV (n=8)**
Chronic antibody mediated rejection	4	2 recipients within one year and 2 whithin 6 months	2
Acute cellular rejection 1A	1	within 6 months	1
Acute humoral rejection	5	all within 2 weeks	4
Acute humoral rejcetion and Acute cellular rejection 2A	1	within 2 weeks	1

## Data Availability

The data used to support the findings of this study are available from the corresponding author upon request.
